# Decoding the neural signatures of valence and arousal from portable EEG headset

**DOI:** 10.3389/fnhum.2022.1051463

**Published:** 2022-12-06

**Authors:** Nikhil Garg, Rohit Garg, Apoorv Anand, Veeky Baths

**Affiliations:** ^1^Institut Interdisciplinaire d'Innovation Technologique (3IT), Université de Sherbrooke, Sherbrooke, QC, Canada; ^2^Laboratoire Nanotechnologies Nanosystèmes (LN2)—CNRS UMI-3463, Université de Sherbrooke, Sherbrooke, QC, Canada; ^3^Institute of Electronics, Microelectronics and Nanotechnology (IEMN), Université de Lille, Lille, France; ^4^Department of Computer Science and Information Systems, BITS Pilani, K K Birla Goa Campus, Goa, India; ^5^Department of Biological Sciences, BITS Pilani, K K Birla Goa Campus, Goa, India; ^6^Cognitive Neuroscience Lab, BITS Pilani, K K Birla Goa Campus, Goa, India

**Keywords:** signal processing, electroencephalography (EEG), machine learning, valence, arousal, emotion, feature extraction, artifact rejection

## Abstract

Emotion classification using electroencephalography (EEG) data and machine learning techniques have been on the rise in the recent past. However, past studies use data from medical-grade EEG setups with long set-up times and environment constraints. This paper focuses on classifying emotions on the valence-arousal plane using various feature extraction, feature selection, and machine learning techniques. We evaluate different feature extraction and selection techniques and propose the optimal set of features and electrodes for emotion recognition. The images from the OASIS image dataset were used to elicit valence and arousal emotions, and the EEG data was recorded using the Emotiv Epoc X mobile EEG headset. The analysis is carried out on publicly available datasets: DEAP and DREAMER for benchmarking. We propose a novel feature ranking technique and incremental learning approach to analyze performance dependence on the number of participants. Leave-one-subject-out cross-validation was carried out to identify subject bias in emotion elicitation patterns. The importance of different electrode locations was calculated, which could be used for designing a headset for emotion recognition. The collected dataset and pipeline are also published. Our study achieved a root mean square score (RMSE) of 0.905 on DREAMER, 1.902 on DEAP, and 2.728 on our dataset for valence label and a score of 0.749 on DREAMER, 1.769 on DEAP, and 2.3 on our proposed dataset for arousal label.

## 1. Introduction

The role of human emotion in cognition is vital and has been studied for a long time with different experimental and behavioral paradigms. Psychology researchers have tried to understand human perception through surveys for a long time. Recently, with the increasing need to learn about human perception, without human biases and conception of various emotions across people (Ekman, 1972), we observe the increasing popularity of neurophysiological recordings and brain imaging methods. Since emotions are triggered almost instantly, Electroencephalography (EEG) is an attractive choice due to its better temporal resolutions and mobile recording devices (Lang, 1995; Moss et al., 2003; Koelstra et al., 2012; Katsigiannis and Ramzan, 2018; Ko et al., 2021; Tuncer et al., 2021).

The algorithmic pipeline of decoding user intentions through neurophysiological signals consists of denoising, pre-processing, feature extraction, electrode and feature selection, and classification. Although there are deep-learning algorithms (Haselsteiner and Pfurtscheller, 2000; Übeyli, 2009; Schirrmeister et al., 2017; Karlekar et al., 2018; Zhou et al., 2018; Jeevan et al., 2019; Jin and Kim, 2020; Tao et al., 2020) which claim to do the frequency decomposition, feature extraction, and classifier training in the hidden layers, their explainability is limited, and amount of training data required is huge. Machine learning with time-domain features performs weighted spatial-temporal averaging of EEG signals with pattern recognition. Feature extraction methods (Ting et al., 2008; Al-Fahoum and Al-Fraihat, 2014; Oh et al., 2014; Zhang et al., 2016) require human effort, and expertise is required in identifying the appropriate features and electrode location depending on the modality, stimulus, recording instrument, and participant. Moreover, current feature extraction and selection method benchmarks (Song et al., 2018; Dar et al., 2020) for emotion recognition are focused on eliciting emotions through video-based stimuli, and the applicability of the proposed methods for static-image elicited emotional response is limited. Most pattern recognition benchmarks (Placidi et al., 2016; Kusumaningrum et al., 2020; Dhingra and Ram Avtar Jaswal, 2021) for decoding human emotions from EEG signals have been performed with research-grade EEG recording systems with large setup times, sophisticated recording setup, and cost. Although a portable EEG headset has a lesser signal-to-noise ratio, its low-cost and easy use makes it an attractive choice for collecting data from a wider population sample and overcoming the problem of insufficient uniform EEG data for algorithmic research.

In this study, first, we propose a protocol for eliciting emotions by presenting selected images from the OASIS dataset (Kurdi et al., 2016) and signal recording through a low-cost, portable EEG headset. Second, we create a pipeline of pre-preprocessing, feature extraction, electrode and feature selection, and classifier for emotional response (Valence and Arousal) decoding and evaluate it for our dataset and two open-source datasets; incremental training to demonstrate the dependence of performance on population sample size is presented. Third, we rank different categories of feature extraction techniques to evaluate the applicability of feature extraction techniques for highlighting the patterns indicative of emotional responses. Moreover, we analyze the electrode importance and rank different brain regions for their importance. The electrodes' relative importance can help explain the significance of different regions for emotion elicitation, lead to optimized electrode configuration while conducting neural-recording studies, and inspire the development of advanced feature extraction techniques for emotional response decoding. Fourth, we ask if we can automate the feature selection and electrode selection techniques for BCI pipeline engineering and validate the procedure with a qualitative and quantitative comparison with neuroscience literature. Importantly, we validate the pipeline for two open-source datasets based on video-based stimuli and recorded signals through the proposed protocol for eliciting emotions through images. The variety of stimuli, recording instruments, and demography of the population sample aids in eliminating bias and rigorous analysis of different pipeline components. Lastly, we publish the proposed pipeline and recorded dataset for the community.

In the past, the scope of using electrophysiological data for emotion prediction has widened and led to standardized 2D emotion metrics of valence and arousal (Russell, 1980) to train and evaluate pattern recognition algorithms. Human brain-recording experiments have been conducted to associate emotion quantitatively with words, pictures, sounds, and videos (Lang, 1995; Lane et al., 1999; Gerber et al., 2008; Eerola and Vuoskoski, 2011; Leite et al., 2012; Moors et al., 2013; Warriner et al., 2013; Kurdi et al., 2016; Mohammad, 2018). EEG frequency band is dominant during different roles, corresponding to various emotional, and cognitive states (Klimesch et al., 1990; Klimesch, 1996, 1999, 2012; Bauer et al., 2007; Berens et al., 2008; Jia and Kohn, 2011; Kamiński et al., 2012). Besides using energy spectral values, researchers use many other features such as frontal asymmetry, differential entropy and indexes for attention, approach motivation and memory. “Approach” emotions, such as happiness, are associated with left hemisphere brain activity, whereas “withdrawal,” such as disgust, emotions, are associated with right hemisphere brain activity (Davidson et al., 1990; Coan et al., 2001). The left-to-right alpha activity is therefore used for approach motivation. The occipito-parietal alpha power has been found to have correlations with attention (Smith and Gevins, 2004; Misselhorn et al., 2019). Fronto-central increase in theta and gamma activities has been proven essential for memory-related cognitive functions (Shestyuk et al., 2019). Differential entropy combined with asymmetry gives out features such as differential and rational asymmetry for EEG segments are some recent developments as forward-fed features for neural networks (Duan et al., 2013; Torres et al., 2020).

In an attempt to classify emotions using EEG signals, many time-domain, frequency-domain, continuity, complexity (Gao et al., 2019; Galvão et al., 2021), statistical, microstate (Lehmann, 1990; Milz et al., 2016; Shen X. et al., 2020), wavelet-based (Jie et al., 2014), and Empirical (Patil et al., 2019; Subasi et al., 2021) features extraction techniques have been proposed. We have summarized the latest studies using EEG to recognize the emotional state in Table 1.

**Table 1:** Table summarizing various machine learning algorithms and features used to classify emotions on various datasets, reported with accuracy.

**Dataset**	**Classifier**	**Feature extraction method**	**Accuracy (%)**	**References**
DEAP	kNN	Gray-level co-occurrence matrix; Spectral power density	79.58 (Average)	Jadhav et al., 2017
DEAP	kNN	Relative power energy; Logarithmic relative power energy; Absolute logarithmic; Relative power energy	67.51, 68.55, 65.10 (VAD)	Verma and Tiwary, 2017
DEAP	SVM	Hjorth parameters; Entropy; Power of frequency bands; RASM; DASM; Energy of frequency bands using wavelets	65.72 (10 fold CV); 65.92 (LOO-CV)	Khateeb et al., 2021
DEAP	GNB	Spectral power; Spectral power differential asymmetry	61.6, 64.7, 61.8 (VAD)	Koelstra et al., 2012
Video clips	kNN	Absolute logarithmic Recoursing; Energy Efficiency of alpha, beta, and gamma bands decomposed using db4 wavelet function	83.26	Murugappan et al., 2010
Movie clips	SVM	Power spectrum and wavelet decomposition of frequency bands; Entropy exponent; Katz fractal dimension; Feature smoothening using LDS; Feature reduction using PCA, LDA, and CFS	87.53 (Best accuracy)	Wang X. et al., 2011
DEAP	2k-NN	Spectral power of frequency bands; Spectral power difference of symmetric electrodes; Histogram parameters of segment level probability vectors; Dirichlet distribution parameters	76.9, 68.4, 73.9, 75.3 (VADL)	Wang et al., 2014

This paper is organized as follows. Section 2.1 describes the three datasets used for our analysis. The theoretical background and the details of pre-processing steps (referencing, filtering, motion artifact, and rejection and repair of bad trials) are discussed in Section 2.2. Section 2.3 addresses the feature extraction details and provides an overview of the features extracted. Section 2.4 describes the feature selection procedure adopted in this work. Section 3 presents our experiments and results. This is followed by Section 4 for discussion of experiments performed and results obtained in this work. Finally, Section 5 summarizes this work's conclusion and future scope.

## 2. Materials and methods

### 2.1. Datasets

#### 2.1.1. OASIS EEG dataset

##### 2.1.1.1. Stimuli selection

**Figure 1:**
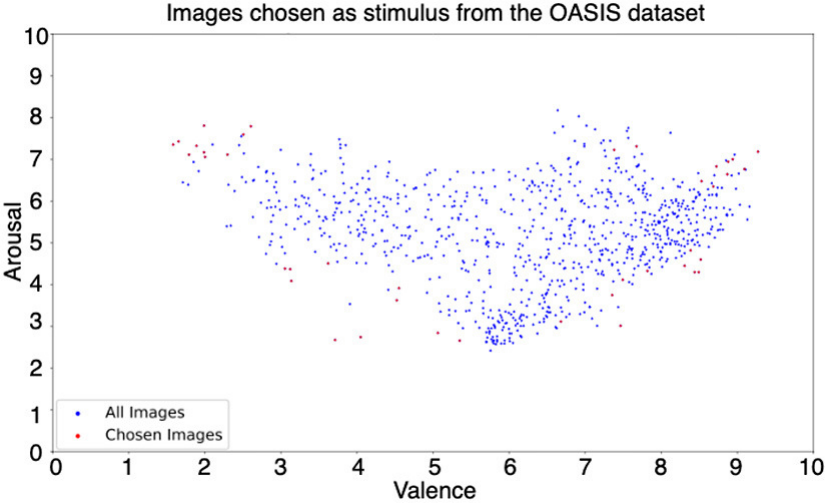
Valence and arousal ratings of OASIS dataset. Valence and arousal ratings of the entire OASIS (Kurdi et al., 2016) image dataset (blue) and the images selected for our experiment (red). The images were selected to represent each quadrant of the 2D space.

The OASIS image dataset (Kurdi et al., 2016) consists of a total of 900 images from various categories, such as natural locations, people, events, and inanimate objects with various valence and arousal elicitation values. Out of 900 images, 40 were selected to cover the valence and arousal rating spectrum, as shown in Figure 1.

##### 2.1.1.2. Participants and device

The experiment was conducted in a closed room, with the only light source being the digital 21” Samsung 1,080 p monitor. Data was collected from fifteen participants of mean age 22 with ten males and five females using an EMOTIV Epoc EEG headset consisting of 14 electrodes according to the 10–20 montage system at a sampling rate of 128 Hz, and only the EEG data corresponding to the image viewing time was segmented using markers and used for analysis.

The study was approved by the Institutional Ethics Committee of BITS, Pilani (IHEC-40/16-1). All EEG experiments/methods were performed in accordance with the relevant guidelines and regulations as per the Institutional Ethics Committee of BITS, Pilani. All participants were explained the experiment protocol, and written consent for recording the EEG data for research purposes was obtained from each subject.

##### 2.1.1.3. Protocol

The subjects were explained the meaning of valence and arousal before the start of the experiment and were seated at a distance of 80–100 cm from the monitor.

**Figure 2:**
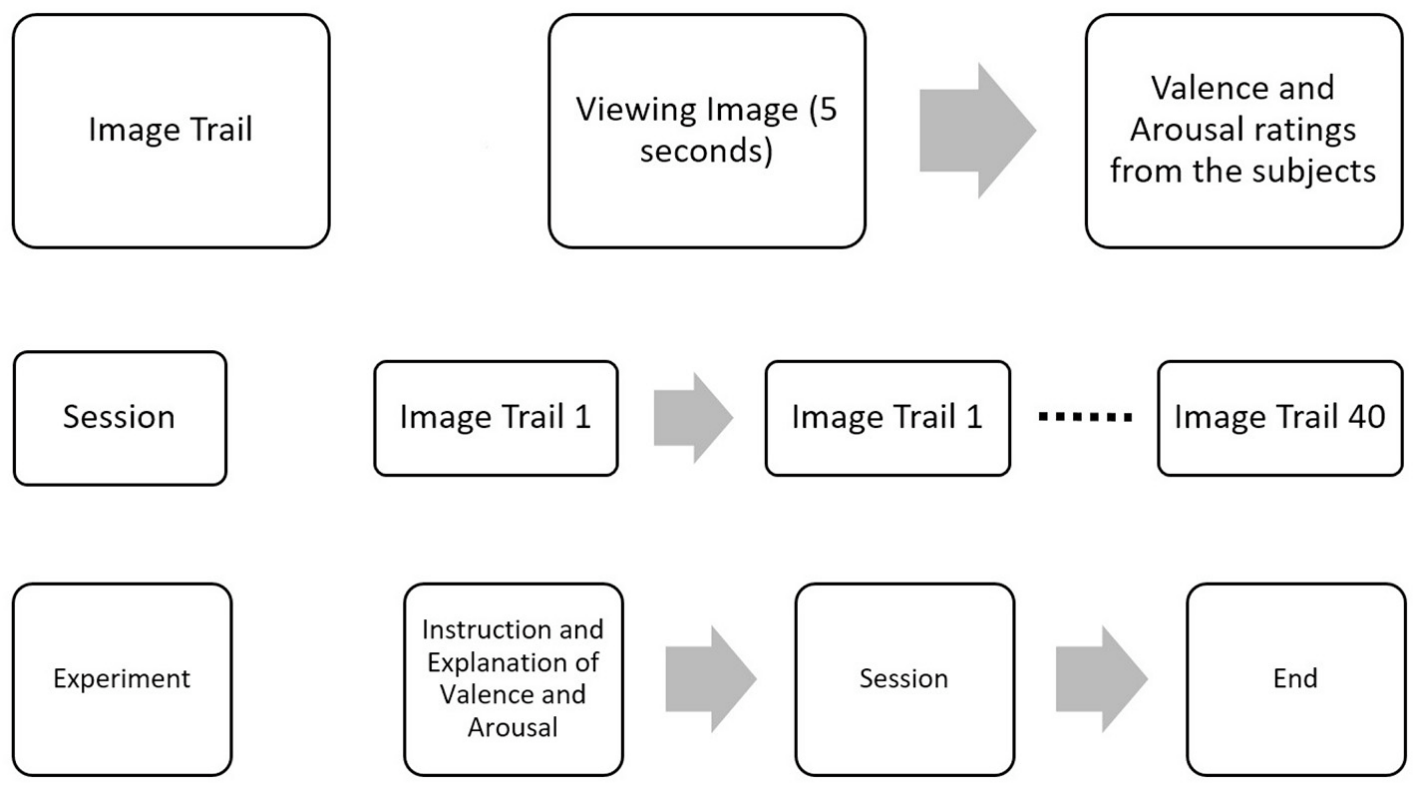
EEG data collection protocol. Experiment protocol for the collection of EEG data. Forty images from the OASIS dataset were shown to elicit emotion in the valence and arousal planes. After presenting each image, ratings were collected from participants.

The images were shown for 5 s through Psychopy (Peirce et al., 2019), and the participants were asked to rate valence and arousal on a scale of 1–10 before proceeding to the next image, as shown in Figure 2. Additionally, the participants' ratings were compared to the original ratings provided in the OASIS image dataset as shown in Supplementary Figure 1, and MSE between the two was 1.34 and 1.39 for valence and arousal, respectively.

#### 2.1.2. DEAP

DEAP dataset (Koelstra et al., 2012) has 32 subjects; each subject was shown 40 music videos one min long. Participants rated each video in arousal, valence, like/dislike, dominance, and familiarity levels. Data was recorded using 40 EEG electrodes placed according to the standard 10–20 montage system. The sampling frequency was 128Hz. This analysis considers only 14 channels (AF3, F7, F3, FC5, T7, P7, O1, O2, P8, T8, FC6, F4, F8, AF4) for the sake of uniformity with the other two datasets.

#### 2.1.3. DREAMER

DREAMER (Katsigiannis and Ramzan, 2018) dataset has 23 subjects; each subject was shown 18 videos at a sampling frequency 128 Hz. Audio and visual stimuli in the form of film clips were employed to elicit emotional reactions from the participants of this study and record EEG and ECG data. After viewing each film clip, participants were asked to evaluate their emotions by reporting the felt arousal (ranging from uninterested/bored to excited/alert), valence (ranging from unpleasant/stressed to happy/elated), and dominance. Data was recorded using 14 EEG electrodes.

### 2.2. Preprocessing

Raw EEG signals extracted from the recording device are continuous, unprocessed signals containing various kinds of noise, artifacts and irrelevant neural activity. Hence, a lack of EEG pre-processing can reduce the signal-to-noise ratio and introduce unwanted artifacts into the data. In the pre-processing step, noise and artifacts presented in the raw EEG signals are identified and removed to make them suitable for analysis in the further stages of the experiment. The following subsections discuss each pre-processing step (referencing, filtering, motion artifact, and rejection and repair of bad trials) in more detail.

#### 2.2.1. Referencing

The average amplitude of all electrodes for a particular time point was calculated and subtracted from the data of all electrodes. This was done for all time points across all trials.

#### 2.2.2. Filtering

A Butterworth bandpass filter of 4th order was applied to filter out frequencies between 0.1 and 40 Hz.

#### 2.2.3. Motion artifact

Motion artifacts were removed by using Pearson Coefficients (Onikura et al., 2015). The gyroscopic data (accelerometer readings) and EEG data were taken corresponding to each trial. Each of these trials of EEG data was separated into its independent sources using Independent Component Analysis (ICA) algorithm. For the independent sources obtained corresponding to a single trial, Pearson coefficients were calculated between each source signal and each axis of accelerometer data for the corresponding trial. The mean and standard deviations of Pearson coefficients were then calculated for each axis obtained from overall sources. The sources with Pearson coefficient 2 standard deviations above the mean for any one axis were high pass filtered for 3 Hz using a Butterworth filter as motion artifacts exist at these frequencies. The corrected sources were then projected back into the original dimensions of the EEG data using the mixing matrix given by ICA.

#### 2.2.4. Rejection and repair of bad trials

Auto Reject is an algorithm developed by Jas et al. (2017) for rejecting bad trials in Magneto-/Electro- encephalography (M/EEG data), using a cross-validation framework to find the optimum peak-to-peak threshold to reject data.

- We first consider a set of candidate thresholds ϕ.
- Given a matrix of dimensions (epochs × channels × time points) by X ∈ R N×P, where *N* is the number of trials/epochs and P is the number of features. P = Q*T, Q is the number of sensors, and T is the number of time points per sensor.
- The matrix is split into K-folds. Each of the K parts will be considered the training set once, and the rest of the K-1 parts become the test set.
- For each candidate threshold, i.e., for each
$$T_{l}\in \phi$$
  we apply this candidate peak to peak threshold (ptp) to reject trials in the training set known as bad trials, and the rest of the trials become the good trials in the training set.
$$ptp\left(X_{i}\right)=max\left(X_{i}\right)-min\left(X_{i}\right)$$
  where *X*_*i*_ indicates a particular trial.
- A is the peak-to-peak threshold of each trial, *G*_*l*_ is the set of trials whose ptp is less than the candidate threshold being considered
$$A=\left\{ptp\left(X_{i}\right)|i\in train_{k}\right\}$$
$$G_{l}=\left\{i\in train_{k}|ptp\left(X_{i}\right)<T_{l}\right\}$$
- Then, the mean amplitude of the good trials (for each sensor and their corresponding set of time points) is calculated
$$\bar{X}=\frac{1}{N}\overset{N}{\underset{i=1}{\sum }}X_{i}$$
- While the median amplitude of all trials is calculated for the test set ${\tilde{X}}_{val_{k}}$
- Now, the Frobenius norm is calculated for all K folds, giving K errors *e*_*k*_ ∈ *E*; the mean of all these errors is mapped to the corresponding candidate threshold.
$$e_{kl}={\left||{\bar{X}}_{G_{l}}-{\tilde{X}}_{val_{k}}|\right|}_{Fro}$$
- The following analysis was done considering all channels at once; thus, it is known as auto-reject global
- Similar process can be considered where analysis can be done for each channel independently, i.e., data matrix becomes(epochs × 1 × time points) known as the local auto-reject, where we get optimum thresholds for each sensor independently.
- The most optimum threshold is the one that gives the least error
$$T_{*}=T_{l_{*}}\;with\;l_{*}=argmin\;l\frac{1}{K}\overset{K}{\underset{i=1}{\sum }}e_{kl}$$

As bad trials were already rejected in the DEAP and DREAMER datasets, we do not perform automatic trial rejections.

### 2.3. Feature extraction

In this work, the following set of 36 features was extracted from the EEG signal data with the help of EEGExtract library (Saba-Sadiya et al., 2020) for all three datasets:

- Shannon Entropy (S.E.)
- Subband Information Quantity for Alpha [8–12 Hz], Beta [12–30 Hz], Delta [0.5–4 Hz], Gamma [30–45 Hz], and Theta[4–8 Hz] band (S.E.A., S.E.B., S.E.D., S.E.G., S.E.T.)
- Hjorth Mobility (H.M.)
- Hjorth Complexity (H.C.)
- False Nearest Neighbor (F.N.N)
- Differential Asymmetry (D.A., D.B., D.D., D.G., D.T.)
- Rational Asymmetry (R.A., R.B., R.D., R.G., R.T.)
- Median Frequency (M.F.)
- Band Power (B.P.A., B.P.B., B.P.D., B.P.G., B.P.T.)
- Standard Deviation (S.D.)
- Diffuse Slowing (D.S.)
- Spikes (S.K.)
- Sharp spike (S.S.N.)
- Delta Burst after Spike (D.B.A.S.)
- Number of Bursts (N.B.)
- Burst length mean and standard deviation (B.L.M., B.L.S.)
- Number of Suppressions (N.S.)
- Suppression length mean and standard deviation (S.L.M., S.L.S.).

These features were extracted with a 1 s sliding window and no overlap. The extracted features can be categorized into two different groups based on the ability to measure the complexity and continuity of the EEG signal. The reader is encouraged to refer to the work done by Ghassemi (2018) for an in-depth discussion of these features.

#### 2.3.1. Complexity features

Complexity features represent the degree of randomness and irregularity associated with the EEG signal. Different features in the form of entropy and complexity measures were extracted to gauge the information content of non-linear and non-stationary EEG signal data.

##### 2.3.1.1. Shannon entropy

Shannon entropy (Shannon, 1948) is a measure of uncertainty (or variability) associated with a random variable. Let X be a set of finite discrete random variables $X=\left\{x_{1},x_{2},...,x_{m}\right\},x_{i}\in R^{d}$, Shannon entropy, *H*(*X*), is defined as


(1) $$\begin{array}{l} H\left(X\right)=-c\overset{m}{\underset{i=0}{\sum }}p\left(x_{i}\right)\ln\;p\left(x_{i}\right) \end{array}$$

where c is a positive constant and p(*x*_*i*_) is the probability of (*x*_*i*_) (ϵ) X such that:


(2) $$\begin{array}{l} \overset{m}{\underset{i=0}{\sum }}p\left(x_{i}\right)=1 \end{array}$$

Higher entropy values indicate high complexity and less predictability in the system (Phung et al., 2014).

##### 2.3.1.2. Subband information quantity

Sub-band Information Quantity (SIQ) refers to the entropy of the decomposed EEG wavelet signal for each of the five frequency bands (Jia et al., 2008; Valsaraj et al., 2020). In our analysis, the EEG signal was decomposed using a butter-worth filter of order 7, followed by an FIR/IIR filter. This resultant wave signal's Shannon entropy [*H*(*X*)] is the desired SIQ of a particular frequency band. Due to its tracking capability for dynamic amplitude change and frequency component change, this feature has been used to measure the information in the brain (Shin et al., 2006; Kanungo et al., 2021).

##### 2.3.1.3. Hjorth parameters

Hjorth Parameters indicate time-domain statistical properties introduced by Hjorth (1970). Variance-based calculation of Hjorth parameters incurs a low computational cost, making them appropriate for EEG signal analysis. We use complexity and mobility (Das and Pachori, 2021) parameters in our analysis. Horjth mobility signifies the power spectrum's mean frequency or the proportion of standard deviation. It is defined as:


(3) $$\begin{array}{l} \text{Hjort}{\text{h}}_{\text{Mobility}}=\sqrt{\frac{\mathrm{var}\left(\frac{dx\left(t\right)}{dt}\right)}{\mathrm{var}\left(x\left(t\right)\right)}} \end{array}$$

where var(.) denotes the variance operator and *x*(*t*) denotes the EEG time-series signal.

Hjorth complexity signifies the change in frequency. This parameter has been used to measure the signal's similarity to a sine wave. It is defined as:-


(4) $$\begin{array}{l} \text{Hjort}{\text{h}}_{\text{Complexity}}=\frac{\mathrm{Mobility}\left(\frac{dx\left(t\right)}{dt}\right)}{\mathrm{Mobility}\left(x\left(t\right)\right)} \end{array}$$

##### 2.3.1.4. False nearest neighbor

False Nearest Neighbor is a measure of signal continuity and smoothness. It is used to quantify the deterministic content in the EEG time series data without assuming chaos (Kennel et al., 1992; Hegger and Kantz, 1999).

##### 2.3.1.5. Asymmetry features

We incorporate Differential Entropy (DE) (Zheng et al., 2014) in our analysis to construct two features for each of the five frequency bands, namely, Differential Asymmetry (DASM) and Rational Asymmetry (RASM). Mathematically, DE [*h*(*X*)] is defined as:


(5) $$\begin{array}{l} h\left(X\right)=-\int_{-\infty }^{\infty }\frac{1}{\sqrt{2\pi \sigma^{2}}}\exp\frac{{\left(x-\mu \right)}^{2}}{2\sigma^{2}}\log\frac{1}{\sqrt{2\pi \sigma^{2}}}\\ \;\exp\frac{{\left(x-\mu \right)}^{2}}{2\sigma^{2}}dx=\frac{1}{2}\log2\pi e\sigma^{2} \end{array}$$

where *X* follows the Gauss distribution *N*(μ,σ^2^), *x* is a variable and π and exp are constant.

Differential Asymmetry (or DASM) (Duan et al., 2013) for each frequency band was calculated as the difference of differential entropy of each of seven pairs of hemispheric asymmetry electrodes.


(6) $$\begin{array}{l} DASM=h\left(X_{i}^{\text{left}}\right)-h\left(X_{i}^{\text{right}}\right) \end{array}$$

Rational Asymmetry(or RASM) (Duan et al., 2013) for each frequency band was calculated as the ratio of differential entropy between each of seven pairs of hemispheric asymmetry electrodes.


(7) $$\begin{array}{l} RASM=h\left(X_{i}^{\text{left}}\right)/h\left(X_{i}^{\text{right}}\right) \end{array}$$

#### 2.3.2. Continuity features

Continuity features signify the clinically relevant signal characteristics of EEG signals (Hirsch et al., 2013; Ghassemi, 2018). These features have been acclaimed to serve as qualitative descriptors of states of the human brain and are important in emotion recognition.

##### 2.3.2.1. Median frequency

Median Frequency refers to the 50% quantile or median of the power spectrum distribution. Median Frequency has been studied extensively due to its observed correlation with awareness (Schwilden, 1989) and its ability to predict imminent arousal (Drummond et al., 1991). It is a frequency domain or spectral domain feature.

##### 2.3.2.2. Band power

Band power refers to the signal's average power in a specific frequency band. The powers of the delta, theta, alpha, beta, and gamma frequency bands were used as spectral features. Initially, a butter-worth filter of order seven was applied to the EEG signal to calculate band power. IIR/FIR filter was applied further on the EEG signal in order to separate out signal data corresponding to a specific frequency band. The average of the power spectral density was calculated using a periodogram of the resulting signal. Signal Processing sub-module (scipy.signal) of SciPy library (Virtanen et al., 2020) in python was used to compute the band power feature.

##### 2.3.2.3. Standard deviation

Standard Deviation has proved to be an important time-domain feature in past experiments (Panat et al., 2014; Amin et al., 2017). Mathematically, it is defined as the square root of the variance of the EEG signal segment.

##### 2.3.2.4. Diffuse slowing

Previous studies (Boutros, 1996) have shown that diffuse slowing correlates with impairment in awareness, concentration, and memory; hence, it is an important feature for estimating valence/arousal levels from EEG signal data.

##### 2.3.2.5. Spikes

Spikes (Hirsch et al., 2013) refers to the peaks in the EEG signal up to a threshold, fixed at mean + 3 standard deviation. The number of spikes was computed by finding local minima or peaks in EEG signal over seven samples using scipy.signal.find_peaks method from SciPy library (Virtanen et al., 2020).

##### 2.3.2.6. Delta burst after spike

The change in delta activity after and before a spike is computed epoch-wise by adding the mean of seven elements of the delta band before and after the spike, used as a continuity feature.

##### 2.3.2.7. Sharp spike

Sharp spikes refer to spikes which last <70 ms and is a clinically important features in the study of electroencephalography (Hirsch et al., 2013).

##### 2.3.2.8. Number of bursts

The number of amplitude bursts(or simply the number of bursts) constitutes a significant feature (Hirsch et al., 2013).

##### 2.3.2.9. Burst length mean and standard deviation

Statistical properties of the bursts, mean μ and standard deviation σ of the burst lengths, have been used as continuity features.

##### 2.3.2.10. Number of suppressions

Burst Suppression refers to a pattern where high voltage activity is followed by an inactive period and is generally a characteristic feature of deep anesthesia (Ching et al., 2012). We use the number of contiguous segments with amplitude suppressions as a continuity feature with a threshold fixed at 10μ (Saba-Sadiya et al., 2020).

##### 2.3.2.11. Suppression length mean and standard deviation

Statistical properties like mean μ and standard deviation σ of the suppression lengths are used as a continuity feature.

### 2.4. Feature selection

After feature extraction, feature selection is performed to optimize the selection and ranking of features, reduce model complexity, decrease computation time and enhance learning precision. The feature selection step plays a crucial role in eliminating redundant features that do not contribute to model performance while preserving the relevant information of EEG signals. Hence, selecting the correct predictor variables or feature vectors can improve the learning process in any machine learning pipeline. In this work, initially, zero-variance or constant features were eliminated from the set of 36 extracted EEG features using the VarianceThreshold feature selection method using sci-kit learn package (Pedregosa et al., 2011). Next, a subset of 25 features common to all 3 datasets (DREAMER, DEAP, and OASIS EEG) was selected after applying the VarianceThreshold method for further analysis. This was done to validate our approach on a common set of features. The set of 11 features (S.E., F.N.N., D.S., S.K., D.B.A.S., N.B., B.L.M., B.L.S., N.S., S.L.M., S.L.S.) were excluded from further analysis. Hence, we reduce the feature space from a set of 36 extracted features to this subset of 25 features. Corresponding to each feature, a feature matrix of shape [*n*_*c*_, *n*_*s*_] is generated. We append all these feature matrices to create a new matrix of shape [*n*_c_*n_*f*_, *n*_*s*_]. This matrix is inverted to get features as columns for each segment, i.e., a matrix of shape [*n*_*s*_, *n*_c_*n_*f*_] where *n*_*c*_ is the number of channels, *n*_*f*_ is the number of features and *n*_*s*_ is the number of segments. These feature column vectors serve as input for the SelectKBest algorithm for performing feature selection and ranking for all three datasets. SelectkBest (Pedregosa et al., 2011) is a filter-based, univariate feature selection method intended to select and retain first k-best features based on the scores produced by univariate statistical tests. In our work, f_regression was used as the scoring function since valence and arousal are continuous numeric target variables. It uses Pearson correlation coefficient as defined in Equation (8) to compute the correlation between each feature vector in the input matrix, X and target variable, y, as follows:


(8) $$\begin{array}{l} \rho_{i}=\frac{\left(X\left[:,i\right]-\mathrm{mean}\left(X\left[:,i\right]\right)\right)*\left(y-\mathrm{mean}\left(y\right)\right)}{\mathrm{std}\left(X\left[:,i\right]\right)*\mathrm{std}\left(y\right)} \end{array}$$

The corresponding *F*-value is then calculated as:


(9) $$\begin{array}{l} F_{i}=\frac{\rho_{i}^{2}}{1-\rho_{i}^{2}}*\left(n-2\right) \end{array}$$

where *n* is the number of samples.

SelectkBest method then ranks the feature vectors based on F-scores returned by the f_regression method. Higher scores correspond to better features.

### 2.5. Regression and evaluation

#### 2.5.1. Random forest regressor

Random forest is an ensemble estimator that fits many classifying decision trees on various sub-samples of the data set and uses averaging over this ensemble of trees to improve the predictive accuracy and control over-fitting (Pedregosa et al., 2011). Moreover, it has been found to be suitable for high-dimensional data. In this experiment, a random forest regressor was implemented with 100 tree estimators and squared-error criterion as base parameters using the sci-kit learn library.

#### 2.5.2. Evaluation metrics

The following regression evaluation metrics were assessed to gauge the model performance as part of this experiment:

##### 2.5.2.1. Root mean squared error (RMSE)

Root Mean Square Error (RMSE) can be defined as the standard deviation of residual errors as shown in Equation (10). Hence, RMSE estimates the deviation of actual values from the predicted regression line. Lower RMSE corresponds to accurate predictions and smaller residual errors by the model. RMSE is more sensitive toward outliers than MAE since the error difference is squared.


(10) $$\begin{array}{l} RMSE(y,\hat{y})=\sqrt{(\frac{1}{n})\overset{n}{\underset{i=1}{\sum }}{\left({\hat{y}}_{i}-y_{i}\right)}^{2}} \end{array}$$

##### 2.5.2.2. ***R***^**2**^ score

*R*^2^ score is a statistic that denotes the proportion of variance in the dependent variable (*y*) explained by independent variables (*x*) of the machine learning model. Higher values of *R*^2^ score correspond to greater ability of independent variables in explaining the variance in the dependent variable. Since the *R*^2^ score depends on the sample size of the dataset and the number of predictor variables, the *R*^2^ score is not meaningfully comparable across datasets of different dimensionality (MAR, 2021). *R*^2^ score can be computed as:


(11) $$\begin{array}{l} R^{2}\left(y,ŷ\right)=1-\frac{\sum_{i=1}^{n}{\left(y_{i}-{ŷ}_{i}\right)}^{2}}{\sum_{i=1}^{n}{\left(y_{i}-ȳ\right)}^{2}} \end{array}$$

##### 2.5.2.3. Mean absolute error (MAE)

Mean Absolute Error or *l*1 loss is the mean of the absolute difference between the predicted value ($\hat{y_{i}}$) and the actual value (*y*_*i*_) of the dependent variable as shown in Equation (12). MAE is a popular linear regression metric that uses the same scale of the observed value. Like RMSE, MAE is also a negatively oriented metric; thus, lower values correspond to more accurate predictions by the model.


(12) $$\begin{array}{l} \mathrm{MAE}\left(y,ŷ\right)=\frac{1}{n_{\text{samples}}}\overset{n_{\text{samples}}-1}{\underset{i=0}{\sum }}|y_{i}-{ŷ}_{i}| \end{array}$$

##### 2.5.2.4. Explained variance (EV)

Explained variance is a part of total variance that acts as a measure of discrepancy between the model and actual data. EV is different from the *R*^2^ score in computation as it does not account for systematic offset and uses biased variance to explain the spread of data points. Hence, if the mean error of the predictor is unbiased, the EV score and *R*^2^ score should become equal. EV can be calculated as:


(13) $$\begin{array}{l} \text{EV}\left(y,ŷ\right)=1-\frac{\mathrm{Var}\left\{y-ŷ\right\}}{\mathrm{Var}\left\{y\right\}} \end{array}$$

where Var{θ} is the variance operator for variable θ.

## 3. Results

### 3.1. Electrodes ranking and selection

**Figure 3:**
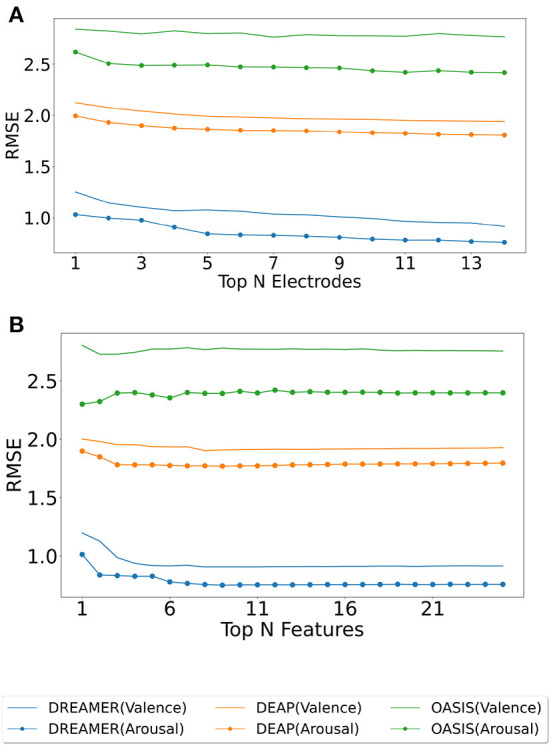
Model evaluation for feature and electrode selection. The random forest regressor was trained on the training set (80%) corresponding to top *N* electrodes (ranked using SelectKBest feature selection method), and RMSE was computed on the test set (20%) for valence (plain) and arousal (dotted) label on DREAMER, DEAP, and OASIS EEG datasets as shown in **(A)**. A similar analysis was performed for top *N* features for DREAMER, DEAP, and OASIS EEG datasets, as shown in **(B)**.

The electrodes were ranked for the three datasets using the SelectKBest method, as discussed in Section 2.4, and the ranks are tabulated for valence and arousal labels in Table 2. To produce a ranking for Top *N* electrodes, feature data for top *N* electrodes were initially considered. The resultant matrix was split in the ratio 80:20 for training and evaluating the random forest regressor model. The procedure was repeated until all 14 electrodes were taken into account. The RMSE values for the same are shown in Figure 3A. It should be noted that, unlike feature analysis, data corresponding to five features each of DASM and RASM was excluded from the Top N electrode-wise RMSE study since these features are constructed using pairs of opposite electrodes.

**Table 2:** Electrode ranking for valence label (V) and arousal label (A) based on SelectKBest feature selection method.

	**DREAMER**		**DEAP**		**OASIS**	

**Electrode**	**A**	**V**	**A**	**V**	**A**	**V**
AF3	10	13	10	8	7	6
AF4	9	11	12	10	8	8
F3	11	10	7	11	5	5
F4	13	14	8	6	6	9
F7	14	12	1	1	1	1
F8	3	5	2	2	4	4
FC5	5	9	14	14	10	7
FC6	6	4	3	4	9	10
O1	12	8	13	7	11	11
O2	8	3	6	9	14	13
P7	4	2	5	3	12	12
P8	7	6	4	5	13	14
T7	1	7	9	13	3	2
T8	2	1	11	12	2	3

### 3.2. Features ranking and selection

Each extracted feature was used to generate its corresponding feature matrix of shape (nbChannels, nbSegments). These feature matrices were then ranked using the SelectKBest feature selection method. Initially, a feature matrix for the best feature was generated. The ranks were tabulated for valence and arousal labels in Table 3. This data was split into 80:20 train-test data; the training data was used to perform regression with Random Forest Regressor, predicted values on test data were compared with actual test labels, and RMSE was computed. In the second run, feature matrices of best and second-best features were combined, data was split into train and test data, the model was trained, and predictions made by the model on test data were used to compute RMSE. This procedure was followed until all the features were taken into account. The RMSE values for the feature analysis procedure, as described above, are shown in Figure 3B.

**Table 3:** Feature ranking for valence label (V) and arousal label (A) based on SelectKBest feature selection method.

**Feature**	**DREAMER**		**DEAP**		**OASIS**	

	**A**	**V**	**A**	**V**	**A**	**V**
B.P.A.	7	5	18	17	25	15
B.P.B.	9	8	8	7	11	13
B.P.D.	22	22	23	22	12	23
B.P.G.	21	18	3	13	5	20
B.P.T.	20	20	19	18	24	17
D.A.	4	11	12	10	16	9
D.B.	12	10	4	4	21	11
D.D.	24	25	16	16	20	21
D.G.	16	16	5	6	14	18
D.T.	13	14	10	9	23	16
H.C.	2	4	20	20	4	4
H.M.	6	3	17	19	1	2
M.F.	14	12	24	25	7	5
R.A.	5	13	21	21	15	12
R.B.	11	9	1	2	19	10
R.D.	23	24	25	24	18	22
R.G.	17	17	2	1	13	19
R.T.	15	15	11	11	22	14
S.E.A.	10	7	13	12	10	24
S.E.B.	3	2	6	5	3	3
S.E.D.	18	19	15	15	8	7
S.E.G.	1	1	9	8	2	1
S.E.T.	19	21	14	14	9	8
S.S.N.	25	23	22	23	17	25
S.D.	8	6	7	3	6	6

### 3.3. Incremental learning

As given by the feature analysis described above, the best features were used to generate a feature matrix for valence and arousal for each dataset. The feature matrix was then used to train a random forest regressor as part of the incremental learning algorithm.

**Figure 4:**
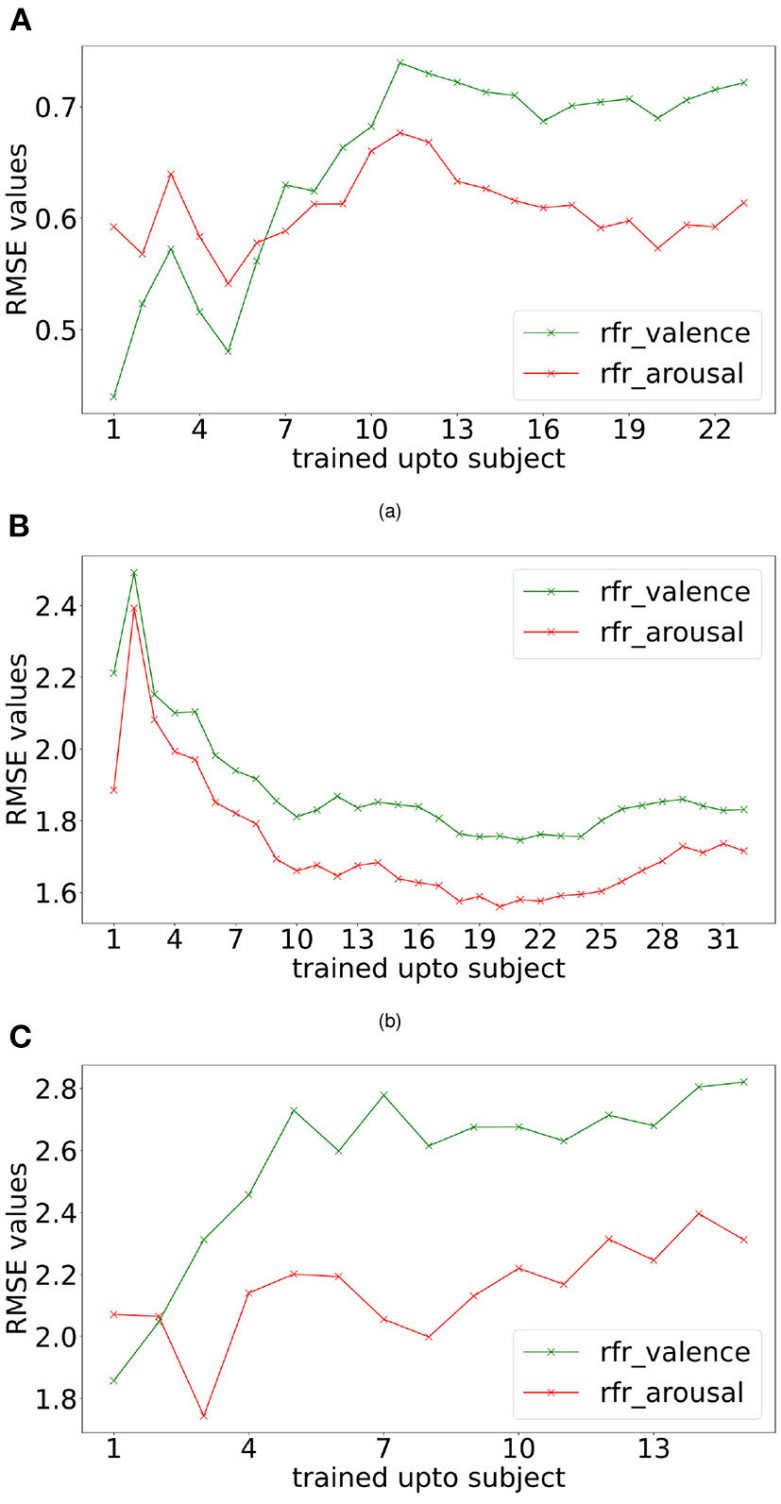
Incremental learning performance. Valence and arousal RMSE readings were obtained with incremental learning for DREAMER **(A)**, DEAP **(B)**, and OASIS EEG **(C)** datasets using random forest regressor (rfr).

Incremental learning was performed based on the collection of subject data. Initially, the first subject data was taken, their trial order shuffled and then split using 80:20 train test size, the model was trained using train split, predictions were made for test data, and next 2nd subject data was taken together with the 1st subject, trial order shuffled, again a train-test split taken and the random forest regressor model was trained using the train split. Predictions were made for the test split. This procedure was repeated until data from all the subjects were used for RMSE computation. RMSE values for each training step, i.e., training data consisted of subject 1 data, then the combination of subject 1, 2 data, then the combination of subject 1, 2, 3 data, and so on. The plots generated for RMSE values for the individual steps of training are shown in Figure 4.

### 3.4. Leave-one-subject-out cross-validation

**Figure 5:**
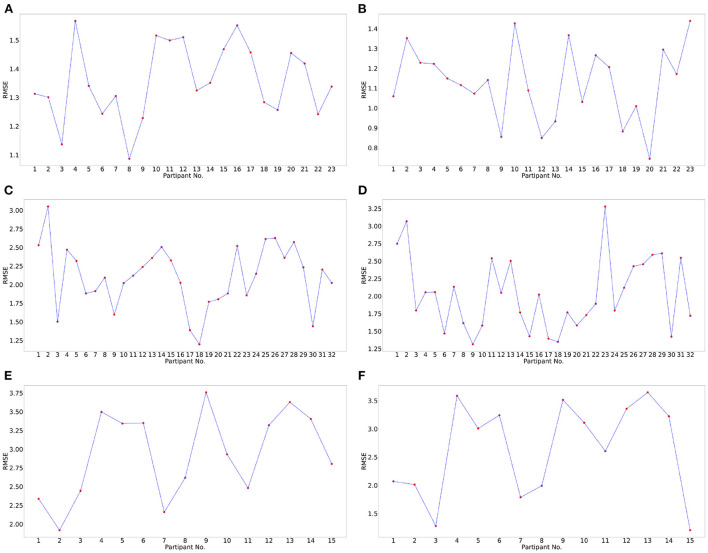
Subject wise performance analysis for valence and arousal labels. Leave-one-subject-out cross-validation performance analysis for valence label for **(A)** DREAMER, **(C)** DEAP, **(E)** OASIS datasets and arousal label for **(B)** DREAMER, **(D)**, DEAP **(F)** OASIS datasets, respectively. In this cross-validation technique, one subject was chosen as the test subject, and the models were trained on the data of the remaining subjects.

Subject generalization is a crucial problem in identifying EEG signal patterns. To prevent over-fitting and avoid subject-dependent patterns. We train the model with data from all the subjects except a single subject and evaluate the model on this remaining subject. Hence, the model is evaluated for each subject to identify subject bias and prevent any over-fitting. Also, when building a machine learning model, it is a standard practice to validate the results by leaving aside a portion of data as the test set. In this work, we used the leave-one-subject-out cross-validation technique to avoid participant bias and evaluate the generalization capabilities of the pipeline. Leave-one-subject-out cross-validation is a k-fold cross-validation technique, where the number of folds, k, equals the number of participants in a dataset. The cross-validated RMSE values for the three datasets for all the participants are plotted in Figure 5.

The mean and standard deviation of RMSE values for valence and arousal labels after cross-validation has been summarized in **Table 6**. The best RMSE values lie within the standard deviation range for the leave-one-subject-out cross-validation results. Hence, inferences drawn from them can be validated.

Table 4 indicates that the optimum values for RMSE, *R*^2^ score, MAE, EV obtained on the test set (20%) using the optimum set of features were 0.905, 0.702, 0.500, 0.702 and 0.749, 0.683, 0.399, 0.683 on the DREAMER dataset; 1.902, 0.247, 1.498, 0.247 and 1.769, 0.273, 1.372, 0.273 on the DEAP dataset; and 2.728, 0.042, 2.320, 0.042 and 2.300, 0.236, 1.815, 0.240 on OASIS dataset for valence and arousal, respectively. For leave-one-subject-out cross-validation, we achieved the best RMSE of 1.35, 1.12 on DREAMER, 2.11, 2.02 on DEAP, and 2.93, 2.64 on the OASIS dataset for valence and arousal, respectively as shown in Figure 5.

**Table 4:** Regression evaluation metrics values for valence and arousal labels on the test set (20%) of DEAP, DREAMER, and OASIS datasets for the optimum set of features.

**Dataset**	**Feature**	**RMSE**	***R* ^2^**	**MAE**	**EV**	**Label**
DREAMER	11	0.905	0.702	0.500	0.702	Valence
DREAMER	9	0.749	0.683	0.399	0.683	Arousal
DEAP	8	1.902	0.247	1.498	0.247	Valence
DEAP	9	1.769	0.273	1.372	0.273	Arousal
OASIS	2	2.728	0.042	2.320	0.042	Valence
OASIS	1	2.300	0.236	1.815	0.240	Arousal

## 4. Discussion

### 4.1. Generalization and overfitting

The dimension of the feature vector is dependent on the number of electrodes and features used for training the machine learning model. Training the model with high-dimensional data requires a proportional sample size to avoid over-fitting. Limited training data and participant bias are classic drawbacks of EEG datasets, especially in the case of emotional state recognition. Therefore determination of the optimum number of electrodes and features is a critical step.

#### 4.1.1. Sample size

For analysing the subject generalization capability of the proposed methods, two experiments were conducted: incremental learning, as shown in Figure 4, and leave-one-out cross-validation, as shown in Figure 4 and **Table 6**. As shown in Supplementary Figure 2, the incremental learning (IL) error is lower than leave-one-out cross-validation (LOCV) for most of the participants. For the DEAP dataset (32 participants), the performance improves when increasing the number of participants considered to train the model (Figure 4B). For DREAMER and OASIS EEG datasets, while the performance worsens while increasing the number of participants (Figures 4A,C), the IL performance is substantially better than LOCV performance (Supplementary Figure 2), indicating participant bias is higher in these two datasets. Moreover, IL learning error saturates after 10 participants in DREAMER and 5 participants in the OASIS EEG dataset. Therefore, the model overfits when trained with data from a few subjects and the generalization capabilities of the proposed model scale with sample size.

#### 4.1.2. Length of feature vector

The optimum number (*N*) of electrodes (Table 5) and features (Table 4) are the ones that produce minimum RMSE in during model evaluation, while the increasing N, as shown in Figures 3A,B. In Figure 3A, a general decline could be seen in the error when increasing the number of electrodes, indicating the importance of high electrode density.

**Table 5:** Regression evaluation metrics values for valence and arousal label on the test set (20%) of DEAP, DREAMER, and OASIS dataset for the optimum set of electrodes.

**Dataset**	**Electrode**	**RMSE**	***R* ^2^**	**MAE**	**EV**	**Label**
DREAMER	14	0.914	0.700	0.500	0.700	Valence
DREAMER	14	0.759	0.675	0.406	0.675	Arousal
DEAP	14	1.938	0.219	1.540	0.219	Valence
DEAP	14	1.806	0.237	1.418	0.237	Arousal
OASIS	7	2.765	0.020	2.357	0.020	Valence
OASIS	14	2.417	0.182	1.917	0.185	Arousal

For the number of features, the downward trend saturates, and even reversal could be observed (Figure 3B) when increasing the number of features beyond a limit. This is also indicated in Table 4 with *N* being less than half the total features for all the datasets. Interestingly, the lowest RMSE was observed with just a single feature for decoding arousal for the OASIS EEG dataset. This might be explained by the fact that OASIS EEG data is smaller than the other two datasets, and increasing the feature-length leads to over-fitting.

The subject generalization capabilities of the learned model can be estimated by comparing leave-one-out cross-validation (Table 6) and standard 80-20 split (Table 4). The former error is higher than the latter by 50, 14, and 9% for DREAMER, DEAP, and OASIS EEG datasets, respectively. The number of selected features is also highest for DREAMER and lowest for the OASIS EEG dataset, indicating that more features increase participant bias and hence should be carefully determined.

**Table 6:** Mean and standard deviation (Std. Dev.) of RMSE values for valence and arousal label data after leave-one-subject-out-cross-validation.

**Dataset**	**Label**	**Mean**	**Std. Dev**.
DREAMER	Valence	1.356	0.130
	Arousal	1.126	0.190
DEAP	Valence	2.112	0.416
	Arousal	2.025	0.519
OASIS	Valence	2.933	0.582
	Arousal	2.642	0.845

### 4.2. Electrode placement analysis

As shown in Tables 2, 3, three rankings were obtained from three datasets for each label. For the valence labels, out of the top 25% electrodes, 33% were in the frontal regions (F3, F4, F7, F8, AF3, AF4, FC5, FC6), 33% in the temporal regions (T8, T7), 22% the parietal regions (P7, P8), and 11% in the occipital regions (O1, O2). Of the top 50% electrodes, 57% were in the frontal regions, 19% in the temporal regions, 19% in the parietal regions, and 4% in the occipital regions.

For the arousal labels, out of the top 25% electrodes, 55% were in the frontal regions, and 44% in the temporal regions. Of the top 50% electrodes, 57% were in the frontal regions, 19% in the temporal regions, 19% in the parietal regions, and 4% in the occipital regions.

Therefore, the frontal region was the most significant brain region for recognizing valence and arousal, followed by the temporal, parietal, and occipital. This is in accordance with previous works on EEG channel selection (Alotaiby et al., 2015; Shen J. et al., 2020).

### 4.3. Feature analysis

The optimum set of features was obtained using feature rankings and model evaluation results present in Tables 3, 4, respectively. For the DREAMER dataset, this set was observed to be (S.E.G, S.E.B, H.M, H.C., B.P.A, S.D, S.E.A, B.P.B, R.B, D.B, D.A) for valence and (S.E.G, H.C, S.E.B, D.A, R.A, H.M, B.P.A, S.D, B.P.B) for arousal respectively. The minimum RMSE values obtained using these optimal features on the DREAMER dataset were 0.905 and 0.749 for valence and arousal dimensions, respectively, as evident from Table 4. Therefore these features were critical for recognizing emotional states and can be used in future studies to evaluate classifiers like Artificial Neural Networks and ensembles.

As shown in Table 3, band power and sub-band information quantity feature for gamma and beta frequency bands performed better in estimating valence and arousal than other frequency bands. Hence the gamma and beta frequency bands are the most critical for emotion recognition (Wang X.-W. et al., 2011; Zheng et al., 2017).

It can be inferred from Table 3 that H.M. was mostly ranked among the top 3 features for predicting valence and arousal labels. Similarly, H.C. was ranked among the top four features. This inference is consistent with the previous studies that claim the importance of time-domain Hjorth parameters in accurate EEG classification tasks (Cecchin et al., 2010; Türk et al., 2017).

In the past, statistical properties like standard deviation derived from the reconstruction of EEG signals have been claimed to be significant descriptors of the signal and provide supporting evidence to the results obtained in this study (Panda et al., 2010; Malini and Vimala, 2016). It was observed that SD was ranked among the top 8 ranks in general.

Additionally, spatial filtering through optimizing the covariance matrices with training data using common spatial patterns (CSP) and Riemannian geometry (Barachant et al., 2011) have been used to aid better classification results (Simar et al., 2020). However, such methods are only applicable for classification tasks, and extension to regression problems is not in the scope of this study. Lastly, the classifier could be further optimized using advanced ensemble learning techniques (Fang et al., 2021) or using deep networks, often referred to as a bag of deep features (Asghar et al., 2019).

## 5. Conclusion and future scope

EEG is a low-cost, noninvasive neuroimaging technique that provides high spatiotemporal information about brain activity, and it has become an indispensable tool for decoding cognitive neural signatures. However, the multi-stage intelligent signal processing method has several indispensable steps like pre-processing, feature extraction, feature selection, and classifier training. In this work, we propose a generalized open-source neural signal processing pipeline based on machine learning to accurately classify emotional index on a continuous valence-arousal plane using these EEG signals. We statistically investigated and validated artifact rejection, automated bad-trial rejection, state-of-the-art spatiotemporal feature extraction techniques, and feature selection techniques on a self-curated dataset recorded from a portable headset in response to the OASIS emotion elicitation image dataset and two open-source EEG datasets. The static images also reduce demographic bias like language and social context and enable generalized benchmarks of different feature extraction for emotional response detection across various recording setups. This published dataset could be used in future studies for intelligent signal processing methods like deep learning, reinforcement learning, and neuromorphic computing. The published simplistic python pipeline would aid researchers in focusing on innovation in specific signal processing steps like feature selection or machine learning without the need to recreate the entire pipeline from scratch. In accordance with neuroscience literature, our proposed system could identify the optimum set of electrodes and features that produce minimum RMSE during emotion classification for a given dataset. It also validated the claim that beta and gamma frequency bands are more effective than others in emotion classification. The OASIS EEG dataset collection was limited to 15 participants due to the COVID-19 pandemic. In future, we plan to collect the data for at least 40 participants to draw stronger inferences. Future work would also include the analysis of end-to-end neural networks and transfer learning for emotion recognition. The published dataset can further advance machine learning systems for emotional state detection with data recorded from portable headsets. The published EEG processing pipeline of artifact rejection, feature extraction, feature ranking, feature selection, and machine learning could be expanded and adapted for processing EEG signals in response to a variety of stimuli.

## Data availability statement

The code supporting this study is made publicly available at https://github.com/rohitgarg025/Decoding_EEG. The OASIS EEG dataset is published at https://zenodo.org/record/7332684#.Y3b_Dt9OlhE.

## Ethics statement

The studies involving human participants were reviewed and approved by Institutional Ethics Committee of BITS, Pilani (IHEC-40/16-1). The patients/participants provided their written informed consent to participate in this study.

## Author contributions

NG and VB conceptualized the research. RG, NG, and AA performed the experiments and analyzed the data. VB supervised the study. NG, RG, AA, and VB approved and contributed to writing the manuscripts. All authors contributed to the article and approved the submitted version.

## Funding

This work was supported by the Department of Science and Technology, Government of India, vide Reference No: SR/CSI/50/2014(G) through the Cognitive Science Research Initiative (CSRI). NG acknowledge financial support from the EU: ERC-2017-COG project IONOS (GA 773228).

## Conflict of interest

The authors declare that the research was conducted in the absence of any commercial or financial relationships that could be construed as a potential conflict of interest.

## Publisher's note

All claims expressed in this article are solely those of the authors and do not necessarily represent those of their affiliated organizations, or those of the publisher, the editors and the reviewers. Any product that may be evaluated in this article, or claim that may be made by its manufacturer, is not guaranteed or endorsed by the publisher.
