# Supplementary material for: Decoding the neural signatures of valence and arousal from portable EEG headset
Source: Front Hum Neurosci. 2022 Dec 6;16:1051463. doi: 10.3389/fnhum.2022.1051463 (PMC9764010; doi:10.3389/fnhum.2022.1051463)
Supplement: Supplementary file 1 [file Data_Sheet_1.PDF]

Supplementary Material

1 PARTICIPANT BIAS ANALYSIS

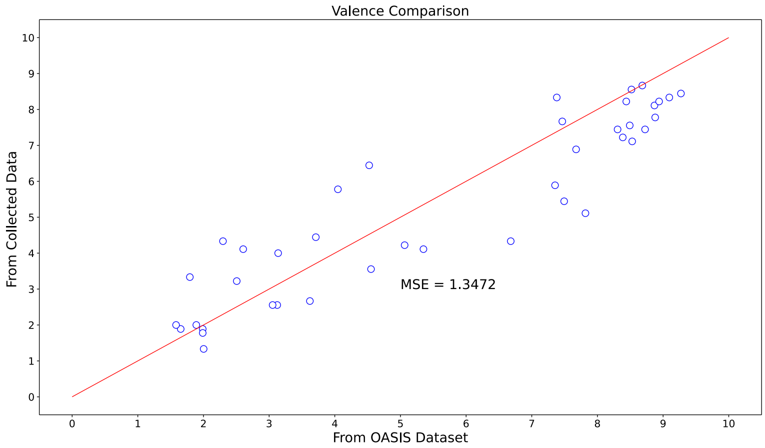

(a)

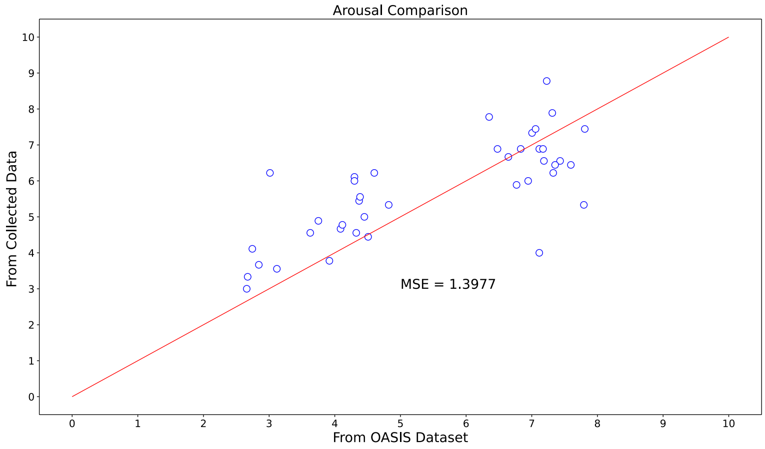

(b)

Figure S1. Comparison of actual and self-reported valence and arousal ratings. Valence (a) and arousal (b) ratings reported by the participants during the EEG data collection and ratings from the OASIS image dataset.

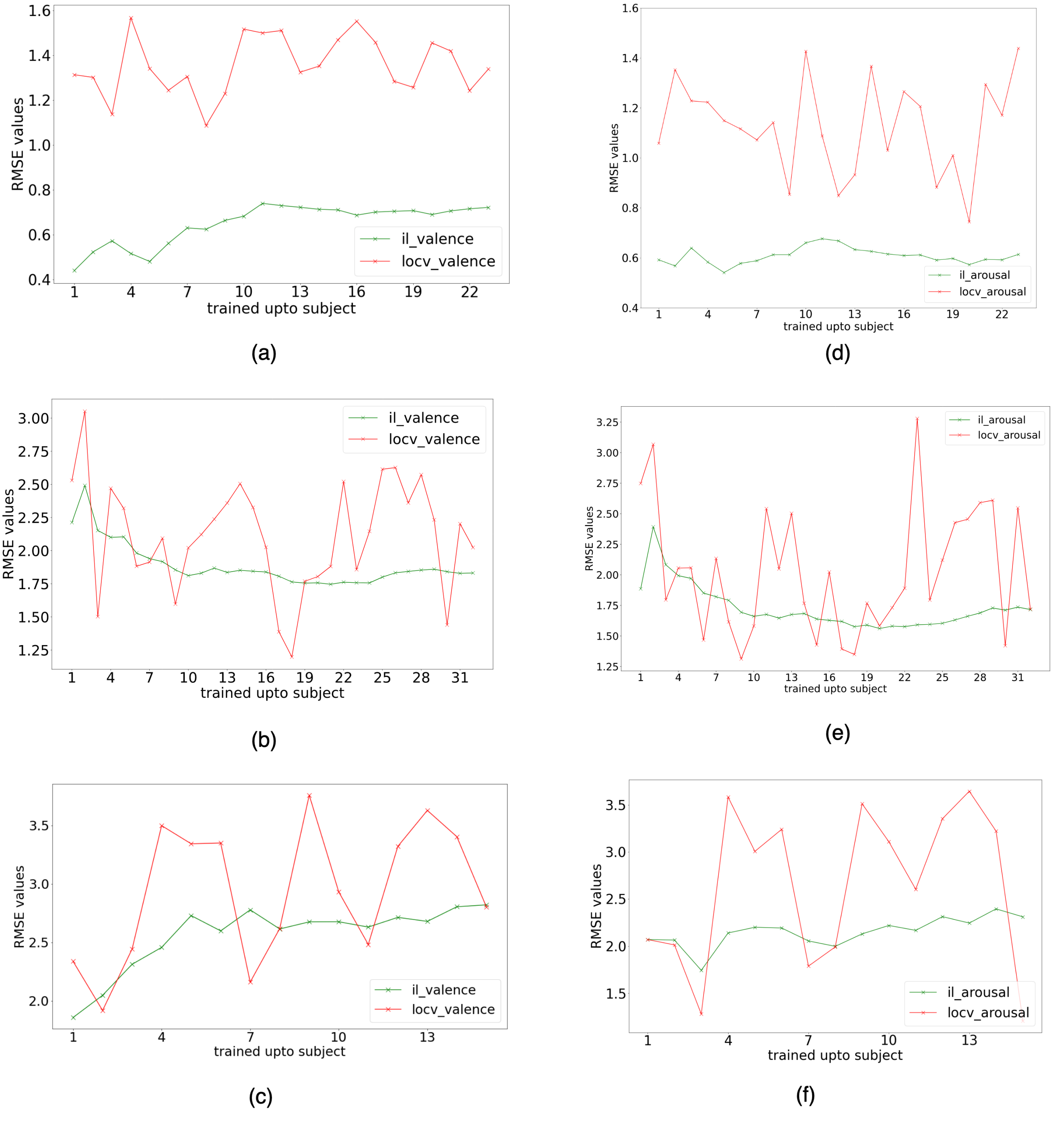

Figure S2. Comparison of incremental learning (il) and leave-one-out-cross-validation (locv) performance for valence label for DREAMER (a), DEAP (b) and OASIS EEG (c) datasets, and for arousal label for DREAMER (d), DEAP (e), and OASIS EEG (f) dataset.
